# Low latency carbon budget analysis reveals a large decline of the land carbon sink in 2023

**DOI:** 10.1093/nsr/nwae367

**Published:** 2024-10-22

**Authors:** Piyu Ke, Philippe Ciais, Stephen Sitch, Wei Li, Ana Bastos, Zhu Liu, Yidi Xu, Xiaofan Gui, Jiang Bian, Daniel S Goll, Yi Xi, Wanjing Li, Michael O'Sullivan, Jefferson Goncalves De Souza, Pierre Friedlingstein, Frédéric Chevallier

**Affiliations:** Department of Earth System Science, Tsinghua University, Beijing 100084, China; Faculty of Environment, Science and Economy, University of Exeter, Exeter EX4 4QF, United Kingdom; Laboratoire des Sciences du Climat et de l'Environnement, University Paris Saclay CEA CNRS, Gif sur Yvette 91191, France; Faculty of Environment, Science and Economy, University of Exeter, Exeter EX4 4QF, United Kingdom; Department of Earth System Science, Tsinghua University, Beijing 100084, China; Institute for Earth System Science and Remote Sensing, Leipzig University, Leipzig 04103, Germany; Department of Biogeochemical Integration, Max Planck Institute for Biogeochemistry, Jena 07745, Germany; Department of Earth System Science, Tsinghua University, Beijing 100084, China; Laboratoire des Sciences du Climat et de l'Environnement, University Paris Saclay CEA CNRS, Gif sur Yvette 91191, France; Machine learning group, Microsoft research, Beijing 100080, China; Machine learning group, Microsoft research, Beijing 100080, China; Laboratoire des Sciences du Climat et de l'Environnement, University Paris Saclay CEA CNRS, Gif sur Yvette 91191, France; Laboratoire des Sciences du Climat et de l'Environnement, University Paris Saclay CEA CNRS, Gif sur Yvette 91191, France; Department of Earth System Science, Tsinghua University, Beijing 100084, China; Faculty of Environment, Science and Economy, University of Exeter, Exeter EX4 4QF, United Kingdom; Faculty of Environment, Science and Economy, University of Exeter, Exeter EX4 4QF, United Kingdom; Faculty of Environment, Science and Economy, University of Exeter, Exeter EX4 4QF, United Kingdom; Laboratoire de Météorologie Dynamique, IPSL, CNRS, ENS, Université PSL, Sorbonne Université, École Polytechnique, Paris 75005, France; Laboratoire des Sciences du Climat et de l'Environnement, University Paris Saclay CEA CNRS, Gif sur Yvette 91191, France

**Keywords:** Global Carbon Budget, El Niño 2023, artificial intelligence emulators of models

## Abstract

In 2023, the CO_2_ growth rate was 3.37 ± 0.11 ppm at Mauna Loa, which was 86% above that of the previous year and hit a record high since observations began in 1958, while global fossil fuel CO_2_ emissions only increased by 0.6% ± 0.5%. This implies an unprecedented weakening of land and ocean sinks, and raises the question of where and why this reduction happened. Here, we show a global net land CO_2_ sink of 0.44 ± 0.21 GtC yr^−1^, which is the weakest since 2003. We used dynamic global vegetation models, satellite fire emissions, an atmospheric inversion based on OCO-2 measurements and emulators of ocean biogeochemical and data-driven models to deliver a fast-track carbon budget in 2023. Those models ensured consistency with previous carbon budgets. Regional flux anomalies from 2015 to 2022 are consistent between top-down and bottom-up approaches, with the largest abnormal carbon loss in the Amazon during the drought in the second half of 2023 (0.31 ± 0.19 GtC yr^−1^), extreme fire emissions of 0.58 ± 0.10 GtC yr^−1^ in Canada and a loss in Southeast Asia (0.13 ± 0.12 GtC yr^−1^). Since 2015, land CO_2_ uptake north of 20°N had declined by half to 1.13 ± 0.24 GtC yr^−1^ in 2023. Meanwhile, the tropics recovered from the 2015–2016 El Niño carbon loss, gained carbon during the La Niña years (2020–2023), then switched to a carbon loss during the 2023 El Niño (0.56 ± 0.23 GtC yr^−1^). The ocean sink was stronger than normal in the equatorial eastern Pacific due to reduced upwelling from La Niña's retreat in early 2023 and the development of El Niño later. Land regions exposed to extreme heat in 2023 contributed a gross carbon loss of 1.73 GtC yr^−1^, indicating that record warming in 2023 had a strong negative impact on the capacity of terrestrial ecosystems to mitigate climate change.

## Teaser

This work reveals an unprecedented weakening of the land carbon sink in 2023 using low-latency atmospheric inversion, dynamic vegetation models, and AI-powered ocean CO2 flux emulators, highlighting the impact of extreme climate events like Amazon droughts and Canadian wildfires.

## INTRODUCTION

**Figure 1.**
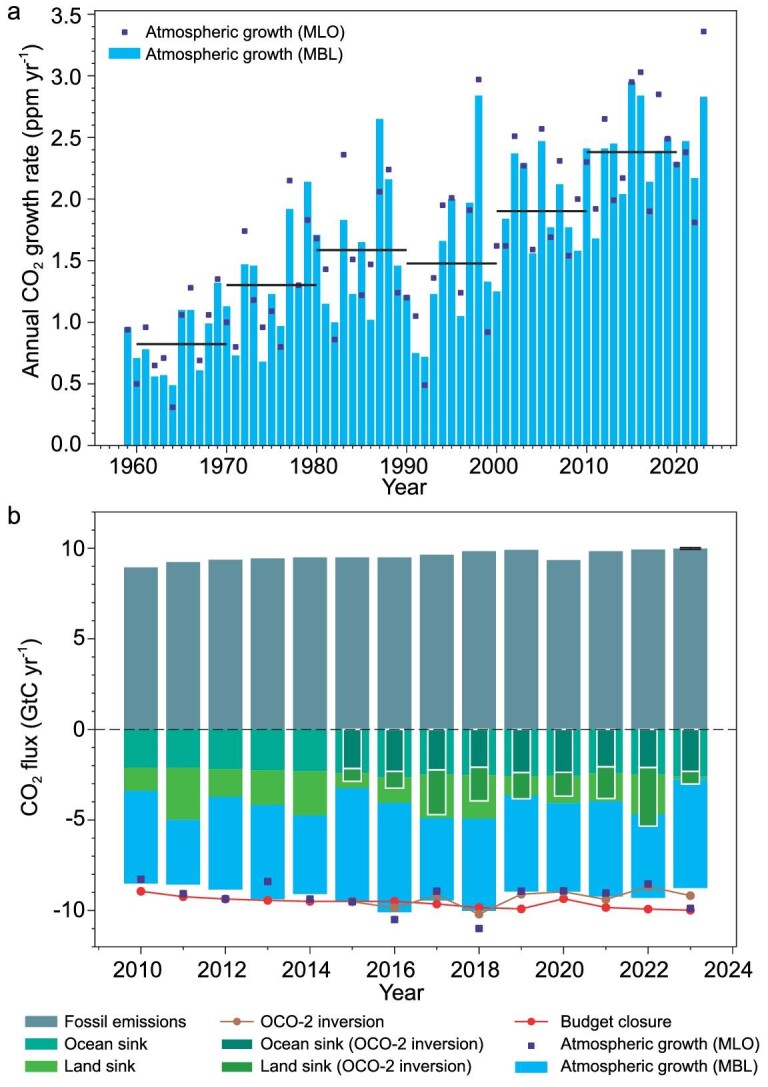
Atmospheric CO_2_ growth rate from 1960 to 2023 and carbon budget from 2010 to 2023. (a) Growth rate from marine boundary layer surface stations (MBL, blue bars) and the Mauna Loa station (MLO, dark blue squares). (b) Global CO_2_ budget obtained with historical fossil fuel and cement CO_2_ emissions, and our estimates of land and ocean sinks in 2023, and the MBL/MLO CO_2_ annual growth rates. Our estimates are based on simulations by emulators of the ocean sink and simulations of the land sink by three dynamic vegetation models forced by low latency climate input data (their mean sink in 2019–2022 being adjusted to equal the median of 16 models used in the latest Global Carbon Budget edition). The ocean and land sinks in the inside bars are from the OCO-2 high-resolution atmospheric inversion. The difference between the stacked bars at the bottom and the red curve (–1 × fossil emissions) is the imbalance of the budget.

The global CO_2_ growth rate (CGR) in the decade of 2013–2022 averaged 2.42 ± 0.08 ppm yr^−1^. In 2023, it increased to a record-high value of 3.37 ± 0.11 ppm yr^−1^ at the Mauna Loa station (MLO) and 2.82 ± 0.08 ppm yr^−1^ from the globally averaged marine boundary layer stations (MBL) [1,2], as shown in Fig. 1a. The growth rate derived from independent OCO-2 satellite observations was 3.03 ± 0.14 ppm yr^−1^ (see ‘Methods’ and Fig. 1). Although, during previous years, the growth rates at MLO and MBL stations have been very close (Fig. 1a), the fact that MLO was higher than MBL in 2023 adds to the uncertainty in understanding the carbon budget of that year. The MLO atmospheric CO_2_ record is influenced by fluxes in Asia and the tropics on timescales of weeks [3]. Therefore, the higher MLO growth rate could be explained by a CO_2_ source anomaly that developed in the tropics late in the year that has not yet fully influenced other remote marine stations. The difference between MLO and MBL extended to mid-2024, which shows that the discrepancy is persisting (see Fig. S1).

To gain insights into the carbon budget in 2023, we assessed global fossil fuel and cement emissions in 2023 from two independent sources: the Carbon Monitor project based on near-real-time (NRT) activity data [4–6] and the approach from the Global Carbon Project based on preliminary energy data with partial global coverage [7]. Both estimates give a small increase of emissions of 0.1%–1.1% (+0.01 to +0.11 GtC yr^−1^) relative to 2022 (Fig. 1b), which only explains a very small fraction of the growth rate anomaly. This implies that natural carbon sinks in the land and oceans must have been drastically reduced in 2023.

A weaker carbon sink in 2023 echoes the impact of extreme warming, globally 0.6°C above the 1991–2020 average and 1.48°C warmer than the 1850–1900 pre-industrial level [8], with extreme summer temperatures [9] and drought in the northern mid-latitudes. The year 2023 was a record high for boreal forest fires in Canada, with 184 961 km^2^ of burned area, which was >2.5 times the previous recorded peak and six times the decadal average [10]. Further, 2023 marked a transition from a long La Niña period since 2020, during which time carbon sinks are expected to be stronger than usual, towards a moderate El Niño that developed after June 2023. The entire year was marked by low water storage on land as observed by the GRACE satellites over most of the northern hemisphere [11], which can cause plant water stress if soil moisture drops below a critical threshold [12]. In the tropics, the Amazon experienced an extreme drought from June to November, whereas tropical Africa remained wetter than normal [13]. On the other hand, the greening of Earth continued in 2023 and reached peak values in the mid-western USA, parts of equatorial Africa, central and south-eastern Europe, southern Brazil and northern Australia [14]. The year 2023 thus provided evidence for a decoupling between global greenness and carbon sinks over land, which deserves a regional analysis of these two variables.

To explain the record-high atmospheric CGR, we developed an integrated approach using top-down and bottom-up estimates of surface CO_2_ exchanges. Over land, we combined three dynamic global vegetation models (DGVMs) [15–21] and a high-resolution atmospheric inversion that assimilated OCO-2 satellite measurements [22]. The three DGVMs have been extensively validated and participated in previous Global Carbon Budget assessments. Here, they were driven by climate reanalysis data that were available with a low latency in order to cover the whole year of 2023 with a slightly modified protocol than that in the global budget assessment (see ‘Methods’). Although we use only three DGVMs, their anomalies for previous years are close to the median of the 21 models that were used in previous carbon budget assessments [7] (see Fig. S2), which gives us confidence that our small sample of fast-track DGVM estimates can still constrain the land sink anomaly for 2023.

For the ocean carbon sink in the bottom-up budget, we used machine-learning emulators of each ocean biogeochemical and the data-driven model that was used for previous years in the Global Carbon Budget assessment [7,23] (Fig. S3). The emulators that are trained by temporal trends and patterns of the original models use as the input data: the atmospheric CO_2_ mixing ratio, sea surface temperature, ice cover, sea surface height, sea level pressure, sea surface salinity, mixed layer depth, wind speed and chlorophyll, which were available in 2023 and allow a projection of the ocean sink in each region for the full year of 2023, as only one ocean data-driven model provided insofar low latency fluxes that covered the first 8 months of the year [24].

For the top-down budget, the availability of OCO-2 observations of atmospheric carbon dioxide column concentration that are updated every 4 months allows us to cover the full year, while most *in situ* surface network measurements are not yet available. Satellite observations from OCO-2 have been shown to provide similar skills in surface CO_2_ flux estimation to assimilations of the more accurate but sparse surface station measurements [22]. Moreover, the OCO-2 data have better coverage than the surface network across the tropics, which is an important advantage during the year 2023 for separating CO_2_ fluxes between the northern hemisphere and the tropics, and investigating flux anomalies between tropical continents, in particular between the Amazon and Southeast Asia that are affected by drought and Central Africa that remained wetter than normal. For the first time, the spatial resolution of our global inversion of ≈1° (see ‘Methods’) better matches that of the DGVM models (0.5°), which allows us to gain more insights into the regional details of CO_2_ fluxes without the usual smoothing effect of inversions. The inversion CO_2_ fluxes were corrected for background natural fluxes that were related to the river loop of the carbon cycle, as in Ref. [25], to provide anthropogenic carbon fluxes that are comparable to those simulated by bottom-up models.

In the northern hemisphere, the occurrence of extreme forest fires in Canada caused massive emissions of CO_2_ during the boreal late spring and autumn. The DGVMs simulate fires from climate conditions and fuel moisture but they have strong weaknesses in capturing extreme forest fires such as those observed in Canada and simulated emissions in the range of 0.05–0.24 GtC y^−1^ during 2023. Therefore, we used emissions based on the burned area and combustion energy observed by satellites from the Global Fire Emissions Database (GFED4.1 s) and the Global Fire Assimilation System (GFAS) to assess fire emissions, and we corrected the DGVM results accordingly (see ‘Methods’). The GFED and GFAS emissions over Canada ranged from 0.48 to 0.68 GtC y^−1^ in 2023.

We do not know whether the OCO-2 inversion that was used for the top-down budget captures the overall effect of fires on the carbon balance of the boreal North America region because the sampling of the atmospheric CO_2_ column by the satellite, with orbits that are spaced 2000 km from each other and clouds that prevent observations, is too coarse to constrain emissions from single fire events. Therefore, three tests were performed in the OCO-2 inversion to estimate the sensitivity of its posterior fluxes to fire emissions: fires were either prescribed with or without vegetation regrowth, or ignored from the prior fluxes (see ‘Methods’). The tests all show very similar boreal CO_2_ flux anomalies in 2023, showing that the inversion, even without prescribed fire emissions in the prior, can still provide a robust diagnostic of the carbon budget of this region (see ‘Methods’).

## RESULTS AND DISCUSSION

### The Global Carbon Budget in 2023

**Figure 2.**
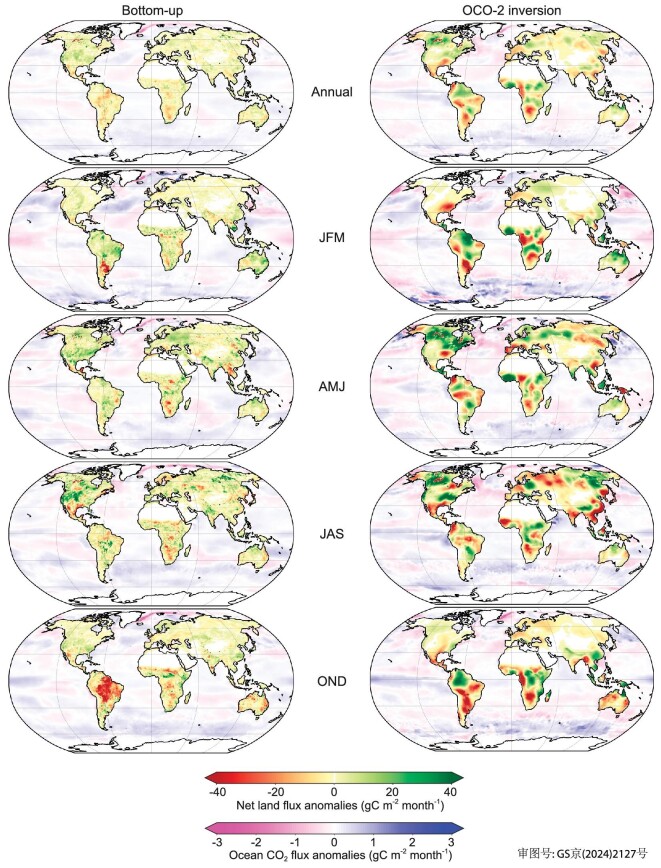
Net land and ocean CO_2_ flux anomalies for each quarter in 2023 compared with the 2015–2022 average for bottom-up models (left column) and the OCO-2 inversion (right column). Positive values represent increased flux from the atmosphere to the land or ocean (carbon sink).

Figure 1 shows the bottom-up carbon budget in 2023 that was obtained from our estimate of fossil fuel emissions (10.20 ± 0.05 GtC yr^−1^) combined with land and ocean carbon sinks from the process model emulators and the top-down budget in which the OCO-2 inversion is used to partition land and ocean sinks. The bottom-up approach does not exactly match the MBL growth rate, with a difference of 0.59 ppm yr^−1^, but it matches the MLO growth rate very well, within 0.04 ppm yr^−1^. This translates into budget imbalances of 1.26 GtC yr^−1^ against MBL and 0.09 GtC yr^−1^ against the MLO growth rates in 2023, which are comparable to the imbalance of the model ensembles that have been used in the Global Carbon Budget over recent years. Note that the inversion that assimilates OCO-2 satellite data produces a global growth rate of 3.03 ppm yr^−1^ (see ‘Methods’), which is in between the MLO and MBL values, suggesting that the MBL stations underestimated the growth rate in 2023, as they did not yet probe the late-year source anomalies in the tropics (Fig. 2).

The net land CO_2_ flux, including land-use-change emissions, decreased to reach a low value of 0.14 ± 0.28 GtC yr^−1^ in 2023 compared with an average of 2.04 GtC yr^−1^ in the period 2010–2022, based on the bottom-up models. This is a record low value compared with previous years since 2003, both in our models (Fig. 1b) and in the models used by the global budget [7]. The OCO-2 inversion diagnosed a small sink of 0.73 ± 0.30 GtC yr^−1^ for the starting El Niño, which was similar to that for the previous El Niño of 2015–2016, which was nevertheless more extreme than the moderate El Niño that started in June 2023. We will need to acquire fluxes until early 2024 to cover the entire period of the current El Niño and compare them to the 2015–2016 event.

In 2023, the ocean carbon sink increased by 0.10 GtC yr^−1^ compared with the year 2022 in our bottom-up approach, to reach a value of 2.60 ± 0.72 GtC yr^−1^. The inversion gives an ocean sink of 2.33 ± 0.20 GtC yr^−1^, in close agreement with our bottom-up model emulators. This increase in the ocean carbon sink was mainly due to the fading La Niña and the developing El Niño, which decreased CO_2_ sources in the tropical Pacific, while high sea surface temperature reduced the sink in the northeastern Atlantic [24].

### Regional anomalies

To gain insights into which regions caused the large drop in the land sink and a coincidental increase in the ocean sink in 2023, we analysed the spatial patterns of flux anomalies for each quarter of the year 2023 in the bottom-up models and in the OCO-2 inversion, using 2015–2022 as a reference period, which corresponds to the period covered by the inversion. The results are displayed in Fig. 2 and quarterly fluxes in 2023 within the distribution of previous years are shown in Fig. S4.

Over the ocean, the most notable increases in carbon uptake were observed in the Pacific Ocean and parts of the Southern Ocean, consistently between the ocean models' emulators and the inversion (Fig. 2). Particularly, the increased uptake was most pronounced in the eastern equatorial Pacific in JAS and OND, consistent with the suppressed upwelling of carbon-rich waters during the developing El Niño [26]. The carbon sinks in the Arctic Ocean, the Indian Ocean and coastal oceans remained relatively unchanged. There is a divergence in the Southern Ocean, where ocean model emulators suggest a slight decrease but the OCO-2 inversion indicates an increase (Fig. S5 and Table S2). On a quarterly basis, the growth of the global ocean sink was mainly observed during the two last quarters of 2023, with the arrival of El Niño conditions.

The regional quarterly land flux anomalies in Fig. 2 indicate large spatial contrasts, which are roughly consistent between the inversion and the bottom-up models (*R*^2^ = 0.33 for land, *R*^2^ = 0.30 for ocean). In general, the inversion shows dipoles of land sources and sinks of larger magnitude than the DGVMs (Fig. S7). The northern land uptake, which usually peaks in summer (JAS) (1.08 GtC month^−1^ in the inversion, 0.52 GtC month^−1^ in the DGVMs north of 20°N during 2015–2022), was lower in 2023, with abnormal sources emerging in Central Europe, western Russia and central America (Fig. 2 and Figs S4 and S6). Yet, the JAS period in 2023 shows a large sink in the northwestern part of North America of 0.11 GtC month^−1^ in the inversion, but not in the DGVMs (0.01 GtC month^−1^), over the region from 180° to 100°W and 40°N to 70°N. The prevalence of a net sink despite extreme fire emissions in Canada suggests that the fire emissions were offset by a region-wide exceptional summer uptake, as corroborated by maximum greening anomalies [14]. As stated above, the OCO-2 inversion JAS sink anomaly in North America was very similar between three inversions with different assumptions about prior fire emissions, suggesting that the OCO-2 data can robustly assess the flux anomalies at this spatial scale (Table S3).

In the fourth quarter of 2023 (OND), the land carbon sink was much lower than the average of previous years. During this quarter, large abnormal carbon losses appeared in the Amazon (–0.14 GtC month^−1^ in the inversion, –0.33 GtC month^−1^ in the DGVMs) and were the largest contributor to the drop in the annual global land sink in 2023. In Africa, the inversion shows an abnormal loss of 0.17 GtC month^−1^ and the DGVMs a loss of 0.07 GtC month^−1^ during OND. Abnormal uptake in central and eastern Africa that experienced wetter conditions was offset by sources in southern Africa [11]. In Southeast Asia, both bottom-up and top-down models show a net sink that approaches zero in OND, ranging from 0.003 to 0.01 GtC month^−1^ (Fig. S4).

### Large land sink decline in 2023 driven by extreme events

**Figure 3.**
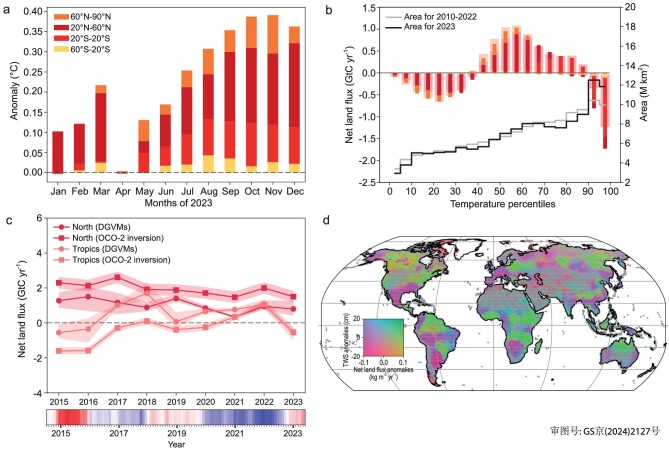
(a) Land temperature warming anomaly each month in 2023 from the reference period 1991–2020 in four large latitude bands. (b) Land CO_2_ fluxes in 2010–2022 (wide bars) and in 2023 (inside bars) for each percentile of land temperature calculated during the reference period 1991–2020. The colors are for the same latitude bands as (a). The land area in each percentile is represented by the gray line in 2010–2022 and by the black line in 2023. (c) Changes in the declining northern land sink and the variations of tropical land flux with sources in 2015–2016 and 2023, from the bottom-up DGVMs and the OCO-2 inversion, during the period from 2015 to 2023. The ENSO index from NOAA Physical Sciences Laboratory (https://www.psl.noaa.gov/enso/mei) is represented at the bottom, with the extreme El Niño of 2015–2016, the La Niña from mid-2020 to mid-2023 and the moderate El Niño of the second half of 2023. (d) Bivariate maps of GRACE TWS anomalies and net land flux anomalies from OCO-2 inversion in 2023 compared with the 2015–2022 average, showing the coordination between carbon flux anomaly and water storage. Positive values in (b) and (c) indicate flux from the atmosphere to the land (carbon sink), while positive values in (d) represent increased land carbon sinks or increased total water storage.

Overall, it is the compounding coincidence of a large abnormal source in the tropics that was contributed by the Amazon drought offsetting higher sinks in central and eastern Africa and Western North America, and a weak summer uptake in the rest of the northern hemisphere that explain the cancellation of the global land sink in 2023 (Fig. 3c and Table S1). Interestingly, Liu *et al.* [27] noted that, in 2021, despite strong La Niña conditions, the land sink was not as large as during previous strong La Niña events and explained this phenomenon by a smaller northern hemisphere uptake that was compensated by a higher tropical sink. Here, in 2023, we see a continuation and an amplification of a weakening northern sink, coinciding with a large abnormal carbon loss in the tropics with the moderate El Niño that arrived in the second half of the year. Since 2015, the northern hemisphere land sink north of 20°N continuously declined by a factor of 2 to reach 1.13 ± 0.24 GtC yr^−1^ in 2023 (mean of bottom-up and top-down) while the tropics recovered from an extreme carbon loss after the 2015–2016 El Niño and remained a sink during the long sequence of wet and cool La Niña years from 2020 to early 2023, then switched to a carbon source during the moderate El Niño in the end of 2023 (Fig. 3c).

**Figure 4.**
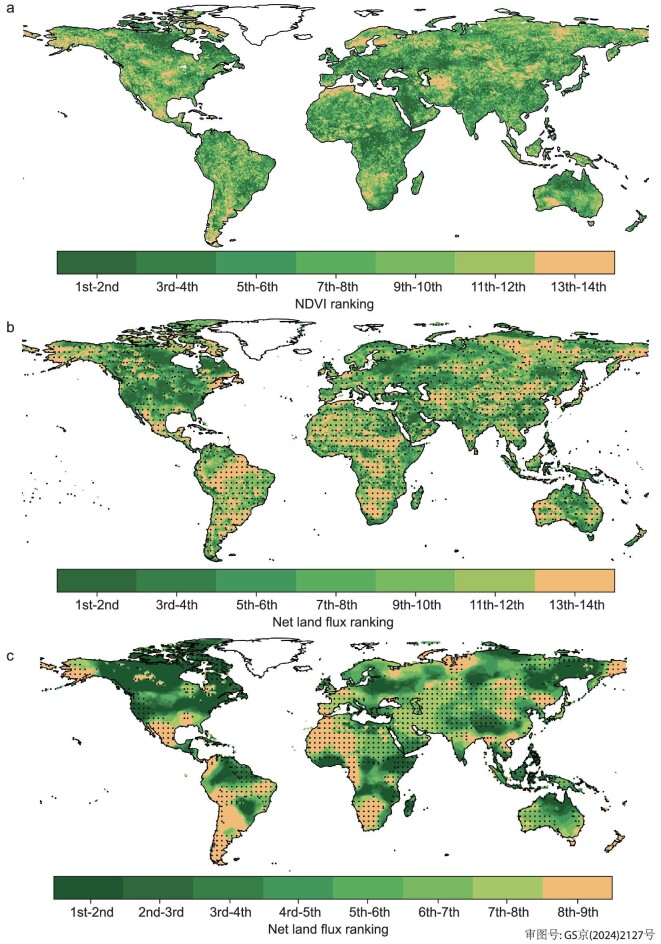
(a) Ranks of the greenness index (NDVI from [43]) in each grid cell during 2010–2023 (rank 1 indicates the highest value among the 14 years). (b) Ranks of the bottom-up DGVM fluxes during the same period (rank 1 indicates the highest sink among the 14 years, rank 14 indicates the smallest sink or the largest source; to better appreciate whether a pixel of low rank is a source or remains a sink, each pixel that was an annual CO_2_ source in 2023 is stippled. (c) Ranks of the OCO-2 inversion fluxes during 2015–2023 (rank 1 indicates the highest sink among the 9 years, rank 9 indicates the smallest sink or the largest source).

Intriguingly, 2023 shows a strong negative relationship between a reduced global land sink and a record-high global greening level [14]. Yet, when looking at the regional scale, we found the expected positive spatial relationships between greening and carbon uptake (Fig. 4). In North America, local burned forest areas where browning was observed have, however, lost a disproportionate amount of carbon compared with the diffuse gains over the large area of non-burned forests with widespread greening. Therefore, it is the fact that carbon losses are very intense over very small disturbed areas where browning is observed that leads to an apparent global decoupling between greening and carbon sinks, but local carbon losses are still associated with browning and gains with greening. A second reason for the local decoupling between greening and carbon sinks is that burned forests recover to a higher greenness very shortly after fires [28], while they recover carbon at a very slow rate [29].

We also found in 2023 that many areas with weaker carbon sinks or higher carbon sources systematically associate with water storage deficits, as observed by the GRACE satellites (Fig. 3d), in water-limited regions. Some regions in which the vegetation is not limited by water availability, such as the Pacific Northwest, still experienced a water deficit and higher carbon sinks in 2023. Thus, care should be taken about systematically expecting that low water availability turns ecosystems into carbon sources—this is only true over regions that lie close to their water limitation point [12].

Given the occurrence of record-breaking temperatures in 2023, we finally analysed the carbon flux anomalies over all grid cells, grouped per percentile intervals of temperature anomalies from the reference period of 1991–2020. The results shown in Fig. 3a and b indicate that extremely hot grid cells (above the 95th percentile) altogether contributed a gross carbon loss of –1.73 GtC yr^−1^ over the globe, including –0.27 GtC yr^−1^ in the northern mid-latitudes (20–60°N) and –1.36 GtC yr^−1^ over the tropics (20°S–20°N). The 95th hottest grid cells account for 29.57% of the global gross carbon loss and have no gross carbon sink in 2023, although they cover only 8.61% of the global land area, showing the disproportionate impact of extremely hot temperatures on negating carbon uptake by the land vegetation.

This result is alarming, as temperatures continue to retain very high values in 2024. It is too early to conclude a durable collapse of the land sink in the aftermath of 2023. Yet, forests that burned in Canada will not completely restore their carbon stocks for the next decades, given that it takes ∼100 years for boral trees to recover their initial biomass [30]. Forests in the wet tropics have proven to be vulnerable to previous extreme droughts, but they were also found to recover quickly, e.g. within a few years in most regions after the severe past El Niño of 2015–2016 [31]. However, forest resilience has been decreasing over time in the Amazon [32]. It is unknown whether a new regime of hotter droughts in the tropics will induce a shift in tree mortality that could turn these critical carbon-rich systems into long-term carbon sources. As per northern forests, it seems that recurrent hot conditions had already begun to weaken their carbon uptake at least since 2021 [27]. The resilience of these systems to droughts and drought-related impacts (insect attacks) combined with future management practices such as harvest rates will determine the near-future stability of the northern sink.

The observation that 2023 had an exceptionally weak land sink despite being only a moderate El Niño constitutes a test bed for Earth System models which lack processes that cause rapid carbon losses, such as extreme fires and climate-induced tree mortality, in their projections, and may thus be too optimistic for estimating the remaining carbon budgets [33]. If very high warming rates continue in the next decade and negatively impact the land sink as they did in 2023, it calls for urgent action to enhance carbon sequestration and reduce greenhouse gas emissions to net zero before reaching a dangerous level of warming at which natural CO_2_ sinks may no longer provide humanity with the mitigation service that they have offered so far by absorbing half of the human-induced CO_2_ emissions.

## METHODS

### Atmospheric CGR

We used the monthly time series of the globally averaged marine surface (MBL) atmospheric CO_2_ concentration that covered the period from January 1979 to February 2024, and the MLO station data from March 1958 to April 2024, both provided by NOAA's Global Monitoring Laboratory (NOAA/GML) [1,2]. The annual MBL growth rate is determined by averaging the most recent December and January months, corrected for the average seasonal cycle, and subtracting the average of the same period in the previous year (https://gml.noaa.gov/ccgg/trends/gl_gr.html). For the MLO data, the annual mean growth rate is estimated by using the average of the most recent November–February months, corrected for the average seasonal cycle, and subtracting the same 4-month average of the previous year centered on 1 January (https://gml.noaa.gov/ccgg/trends/gr.html).

### Global fire CO_2_ emissions

Both the Global Fire Emissions Database version 4.1 including small fire burned area (GFED4.1 s) [34] and the GFAS (https://atmosphere.copernicus.eu/global-fire-monitoring) from Copernicus Atmosphere Monitoring Service (CAMS) are used to derive monthly global fire CO_2_ emissions. GFED4.1 s combines satellite information on fire activity and vegetation productivity to estimate the gridded monthly burned area and fire emissions, and has a spatial resolution of 0.25° × 0.25° [34]. Note that GFED4.1 s fire emissions in 2017 and 2023 are from the beta version. The GFAS assimilates fire radiative power observations from satellites to produce daily estimates of biomass burning emissions. We aggregated the GFAS daily fire emissions into monthly emissions.

### Terrestrial CO_2_ fluxes

Terrestrial carbon fluxes are derived from the mean of three DGVMs, specifically ORCHIDEE, JULES and OCN. The methodology for estimating terrestrial carbon fluxes for the period 2010–2023 aligns with the TRENDY protocol that is used in Global Carbon Budgets [35], albeit with modifications due to the use of ERA5 climate forcing and the fact that land-use forcing is not updated with such short latency. ERA5 forcing has been available since 1940 [36] but preliminary simulations with DGVMs showed some issues with precipitation forcing before 1960. The simulations that were performed here correspond to the S2 experiment, starting from a steady state in 1960, with time-varying climate and CO_2_ forcing, and land use fixed at 2010, as described in [37].

Since the model simulations started from a steady state in the 1960s, they cannot account for the carbon balance before the Industrial Revolution, which leads to an underestimation of the mean trend compared with standard DGVMs. Therefore, we calibrated them by using the 2019–2022 TRENDY DGVMs Simulation 3 from the Global Carbon Budget 2023 [7]. This was done by calculating, for each grid cell, the median of the carbon flux from the TRENDY models during 2015–2022 and the mean of each of the DGVMs used in this study, then subtracting from each DGVM on each grid the difference so that they matched the median flux of the TRENDY models. In other words, our DGVMs were used for predicting interannual anomalies of CO_2_ fluxes.

### Ocean CO_2_ fluxes

Ocean carbon fluxes are derived from a suite of emulators that are based on both biogeochemical models and data-driven models. We updated estimates from five Global Ocean Biogeochemical Models and eight data products that were included in the Global Carbon Budget 2022 to create an NRT framework [23]. This update employs Convolutional Neural Networks and semi-supervised learning techniques to capture the non-linear relationships between model or product estimates and observed predictors. As a result, we obtained an NRT monthly grid-based data set of global surface ocean fugacity of CO_2_ and ocean–atmosphere CO_2_ flux data, extending to December 2023. More details are given in [23] and the Supplementary data.

### Anthropogenic CO_2_ emissions

For the period from 2010 to 2022, we used global fossil fuel and cement CO_2_ emission estimates from the latest edition of the Global Carbon Budget [7]. For 2023, we used the average of emissions from the Carbon Monitor project that were based on NRT activity data from six sectors [4–6] and from an updated estimate by using the same methodology as the Global Carbon Budget [7], i.e. based on energy data that were available by the time of the publication and projections for countries with no available data. The Carbon Monitor data give a global emission of +0.1% compared with 2022, while the Global Carbon Budget approach gives a global emission of 1.1% compared with 2022.

### Atmospheric inversion

We used a global high-resolution atmospheric inversion that was driven by the OCO-2 satellite atmospheric CO_2_ concentration data [38] called CAMS. It can deliver global estimates of weekly greenhouse gas fluxes with a typical 4-month latency, now at a resolution of 0.7° in latitude and 1.4° in longitude. The product used here is an intermediate version between version FT23r3 and the coming FT23r4. It follows the usual production and quality-control process of the CAMS product. It covers the OCO–2 period from 2015 to December 2023, and its mean fluxes and anomalies are close to the median of 14 inversions that were used in previous assessments [7] (see Fig. S8).

The underlying transport model was nudged towards horizontal winds from the ERA5 reanalysis. The inferred fluxes were estimated in each horizontal grid point of the transport model with a temporal resolution of 8 days, separately for daytime and night-time. The prior values of the fluxes combine estimates of (i) gridded monthly fossil fuel and cement emissions (GCP-GridFED version 2023.1 [39]) extended to 2023 following Chevallier *et al.* (2020) [40] by using the emission changes reported by https://carbonmonitor.org/, together with anomalies in retrievals of NO_2_ columns from the Tropospheric Monitoring Instrument (TROPOMI, offline and processing and RPRO when available, van Geffen *et al.*, 2019 [41]); (ii) monthly ocean fluxes (Chau *et al.* 2023 [24], 2024 [42]), 3-hourly (when available) or monthly biomass burning emissions (GFED 4.1 s) described in van der Werf *et al.* (2017) [34] and climatological 3-hourly biosphere–atmosphere fluxes taken as the 1981–2020 mean of a simulation of the ORganizing Carbon and Hydrology In Dynamic EcosystEms model, version 2.2, revision 7262 (ORCHIDEE, Krinner *et al.* 2005 [20]). The variational inversion accounts for spatial and temporal correlations of the prior errors, resulting in a total 1-sigma uncertainty for the prior fluxes over a full year of 3.0 GtC yr^−1^ for the land pixels and of 0.2 GtC yr^−1^ for the marine pixels.

### Land fluxes per percentile of land temperature anomalies from the reference period 1991–2020

We utilized 2 m temperature data spanning 1960–2023 from monthly ERA5 data [36]. Monthly gridded data from 1991–2020 were sorted for each grid cell by temperature, categorized into 20 percentiles. Subsequently, temperature data from 2010–2022 and 2023 were used to identify grid cells that fell within these percentiles, calculating their respective areas. Concurrently, matching grid cells were identified from three NRT DGVMs that were employed in this study to compute land fluxes.

### Ranked greening and net land carbon fluxes

We use Moderate Resolution Imaging Spectroradiometer (MODIS) NDVI data [43] to assess vegetation greenness in 2023. We rank the NDVI data for each grid from 2010 to 2023, obtaining the position of the 2023 NDVI of each grid within this period. For land carbon fluxes, we utilize data from three NRT DGVMs and OCO-2 inversion. We rank the 2023 flux for each grid within the periods 2010–2023 for the DGVMs and 2015–2023 for the OCO-2 inversion.

## Supplementary Material

nwae367_Supplemental_File

## Data Availability

The data from Global Carbon Budget 2023 are available at https://www.icos-cp.eu/science-and-impact/global-carbon-budget/2023. The Carbon monitor fossil fuel emissions data set is available at https://carbonmonitor.org/. The GFED 4.1 s fire emissions data set is available at geo.vu.nl/∼gwerf/GFED/GFED4/. The GFAS fire emissions data set is available at https://atmosphere.copernicus.eu/global-fire-monitoring/. The ERA5 monthly averaged data are available at https://cds.climate.copernicus.eu/cdsapp#!/dataset/reanalysis-era5-single-levels-monthly-means?tab=overview. The Multivariate ENSO index is available at https://www.psl.noaa.gov/enso/mei. The MODIS NDVI data are available at https://lpdaac.usgs.gov/products/mod13c2v006/.
