# Supplementary material for: Low latency carbon budget analysis reveals a large decline of the land carbon sink in 2023
Source: Natl Sci Rev. 2024 Oct 22;11(12):nwae367. doi: 10.1093/nsr/nwae367 (PMC11648560; doi:10.1093/nsr/nwae367)
Supplement: nwae367_Supplemental_File [file nwae367_supplemental_file.pdf]

Supplemental Information for

Low latency carbon budget analysis reveals a large decline of the land carbon sink in 2023

Piyu Ke[1,2], Philippe Ciais[3,*], Stephen Sitch[2], Wei Li[1], Ana Bastos[4,5], Zhu Liu[1], Yidi Xu[3], Xiaofan Gui[6], Jiang Bian[6], Daniel S. Goll[3], Yi Xi[3], Wanjing Li[1], Michael O'Sullivan[2], Jefferson Goncalves de Souza[2], Pierre Friedlingstein[2,7], Frédéric Chevallier[3]

1. Department of Earth System Science, Tsinghua University, Beijing 100084, China
2. Faculty of Environment, Science and Economy, University of Exeter, Exeter EX4 4QF, United Kingdom
3. Laboratoire des Sciences du Climat et de l'Environnement, University Paris Saclay CEA CNRS, Gif sur Yvette 91191, France
4. Institute for Earth System Science and Remote Sensing, Leipzig University, Leipzig 04103, Germany
5. Department of Biogeochemical Integration, Max Planck Institute for Biogeochemistry, Jena 07745, Germany
6. Machine learning group, Microsoft research, Beijing 100080, China
7. Laboratoire de Météorologie Dynamique, IPSL, CNRS, ENS, Université PSL, Sorbonne Université, École Polytechnique, Paris 75005, France

***Corresponding author.** E-mail: philippe.ciais@cea.fr

This PDF file includes:

Supplementary Texts
Supplementary Figures 1 to 8
Supplementary Tables 1 to 3

Supplementary Texts

1. Global annual growth rates of atmospheric CO2 from different atmospheric observations and bottom-up models

We compared the atmospheric CO_2 growth rate from 2010 to 2023 based on in-situ observations from 40 marine boundary layer background stations, calculated by NOAA ESRL[1,2] as the year on year difference between smoothed observations between November-February averaged across all the stations (blue bars), from the Mauna Loa station (purple squares), from the assimilation of global OCO-2 satellite observations of column CO_2 concentration measurements, about 300,000 10-second-averaged retrievals each year, by the inversion used in this study (brown dots), and by the bottom-up approach, that is, not using atmospheric measurements and the growth rate is predicted from the difference between fossil fuel CO_2 emissions minus the land sink from three DGVM models, minus the ocean sink from ocean model emulators (red dots) (Supplementary Figure 1).

2. Maps of monthly air-sea CO_2 fluxes from emulators of biogeochemical and data driven ocean models

We utilized a deep learning technique for near-real-time estimation of oceanic carbon monthly gridded fluxes. This method integrates year, month, latitude, longitude, and nine environmental factors as predictors, targeting predictions for each GOBM model or ocean data product. We use monthly data from 5 GOBMs and 8 data products from the Global Carbon Budget 2022[3], covering data up to the end of 2021. The fCO_2 output from each GOBM or data product is provided at a 1° × 1° monthly resolution. Our predictive variables include a range of biological, chemical, and physical factors typically linked to fluctuations in fCO_2. These variables are sea surface temperature (SST), sea ice fraction (ICE), sea surface salinity (SSS), atmospheric CO_2 mole fraction (xCO_2), mixed-layer depth (MLD), sea surface height (SSH), chlorophyll a (chl a), sea level pressure (SLP), and wind speed. All data are bilinearly interpolated to a 1° × 1° monthly resolution to align with our fCO_2 targets and updated to Dec. 2023. Since the xCO_2 data is only available up to the end of 2022, and to meet the requirement for near-real-time data, we gather global average marine boundary layer surface monthly mean atmospheric CO_2 data updated to Dec. 2023. We use a light gradient boosting machine (LightGBM)[4] model to establish a relationship between the year, month, latitude, longitude, mean atmospheric CO_2 data, and xCO_2. We used data from 1979-2021, divided into training and validation datasets in an 8:2 ratio. Early stopping was implemented with LightGBM, and testing on 2022 data yielded a test RMSE of 1.74, reflecting roughly a 0.5% prediction error. This approach allows us to extend the xCO_2 data to near-real-time.

The data are formatted into a 180x360 grid and subdivided into 18x18 patches for computational efficiency. We trained the model on labeled data points, calculating the Root Mean Square Error (RMSE) between labels and predictions as the supervised loss (LsL_sLs). To ensure prediction stability on unlabeled data, we used pseudo-labeling by predicting with 10% of features removed as pseudo-labels, then predicting again with 30% of features removed, calculating the RMSE as the unsupervised loss (LuL_uLu). The model was updated using the

weighted sum of LsL_sLs and LuL_uLu through backward propagation. Our model architecture combines a multi-layered Convolutional Neural Network (CNN)[5] and linear models[6], designed to process the input data efficiently. The input layer has a dimension of 18x18x13, maintained across all CNN and linear layers. The CNN hidden layers have dimensions of 13, 64, and 64, learning spatial hierarchies, while the linear layers have dimensions of 64, 64, and 1, performing linear transformations for predictions. The output layer has a dimension of 1, representing the predicted oceanic carbon fCO_2 value. CNNs, inspired by human visual perception, consist of Convolutional Layers, Rectified Linear Unit (ReLU) Layers, and Fully Connected Layers, which work together to process and transform input data into predictions. This architecture ensures accurate and efficient prediction of oceanic carbon fluxes.

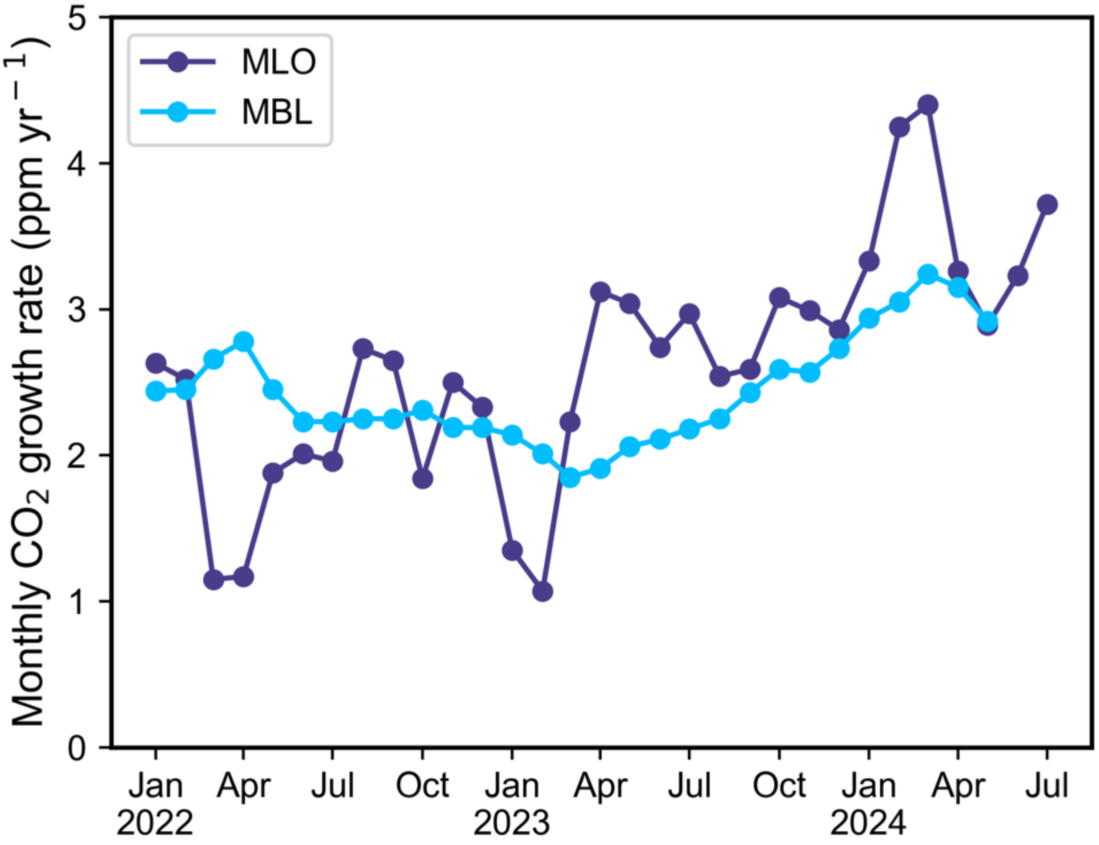

Supplementary Figure 1 Monthly CO2 growth rate from marine boundary layer surface stations (MBL) and the Mauna Loa Observatory (MLO) from January 2022 to July 2024.

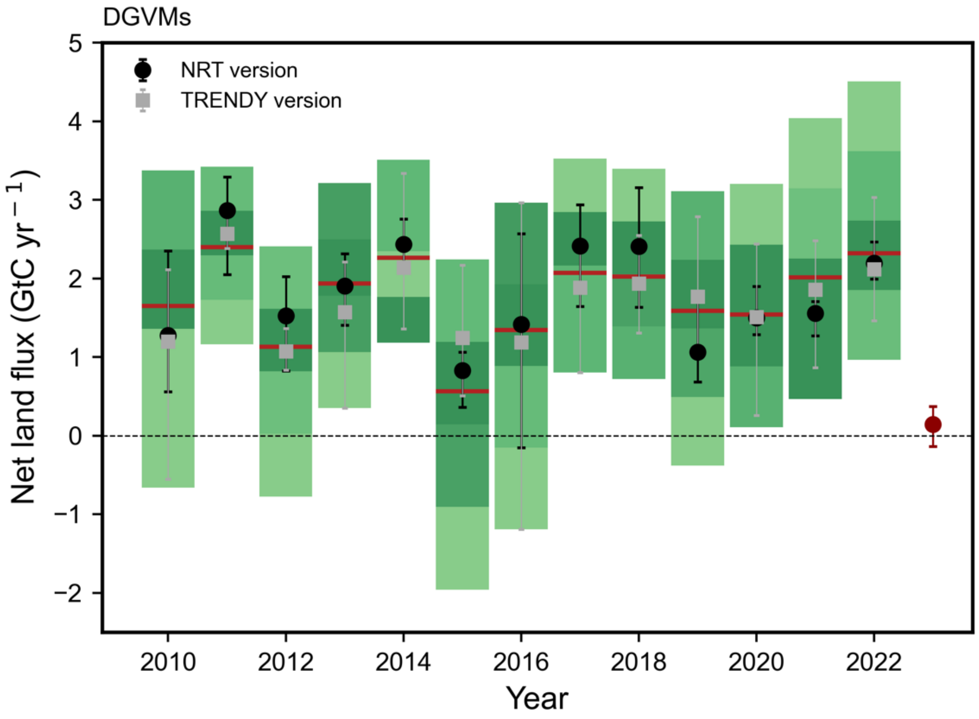

Supplementary Figure 2 Comparison of global net land CO_2 fluxes between the 3 DGVMs used in this study (black dots for NRT version, a red dot for NRT version in 2023, gray squares for TRENDY version) and the distribution of 21 TRENDY models (green bars, with color intensity representing the number of DGVMs falling into each interval) used in latest Global Carbon Budget edition[7]**.** Note that the 3 DGVMs have been biased corrected to have the same net land sink than the average of TRENDY models during 2019-2022, but their anomalies are feely calculated for all years including in 2023. Positive values indicate flux from the atmosphere to the land (carbon sink).

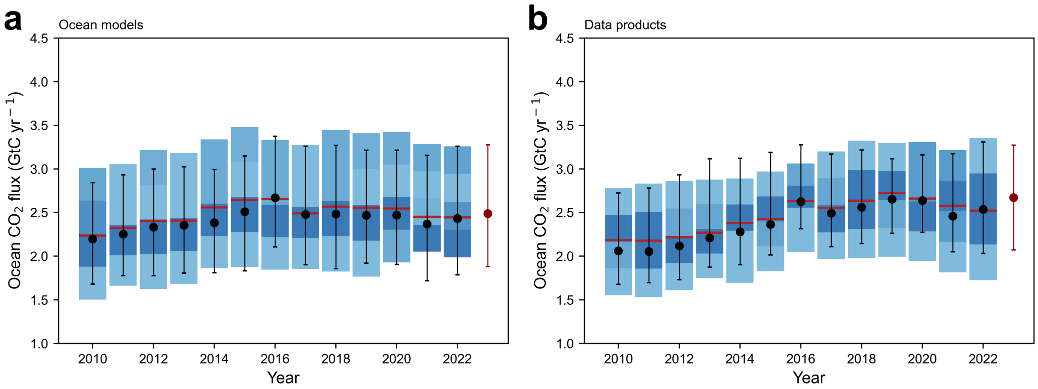

Supplementary Figure 3 Comparison of global ocean CO_2 fluxes between the emulators of ocean models used in this study (black dots for 2010-2022, red dots for 2023) and the distribution of 5 mechanistic ocean biogeochemistry models (a) and 8 data driven models (b) (blue bars, with color intensity representing the number of models falling into each interval) used in latest Global Carbon Budget edition[7]. Positive values indicate flux from the atmosphere to the ocean (carbon sink).

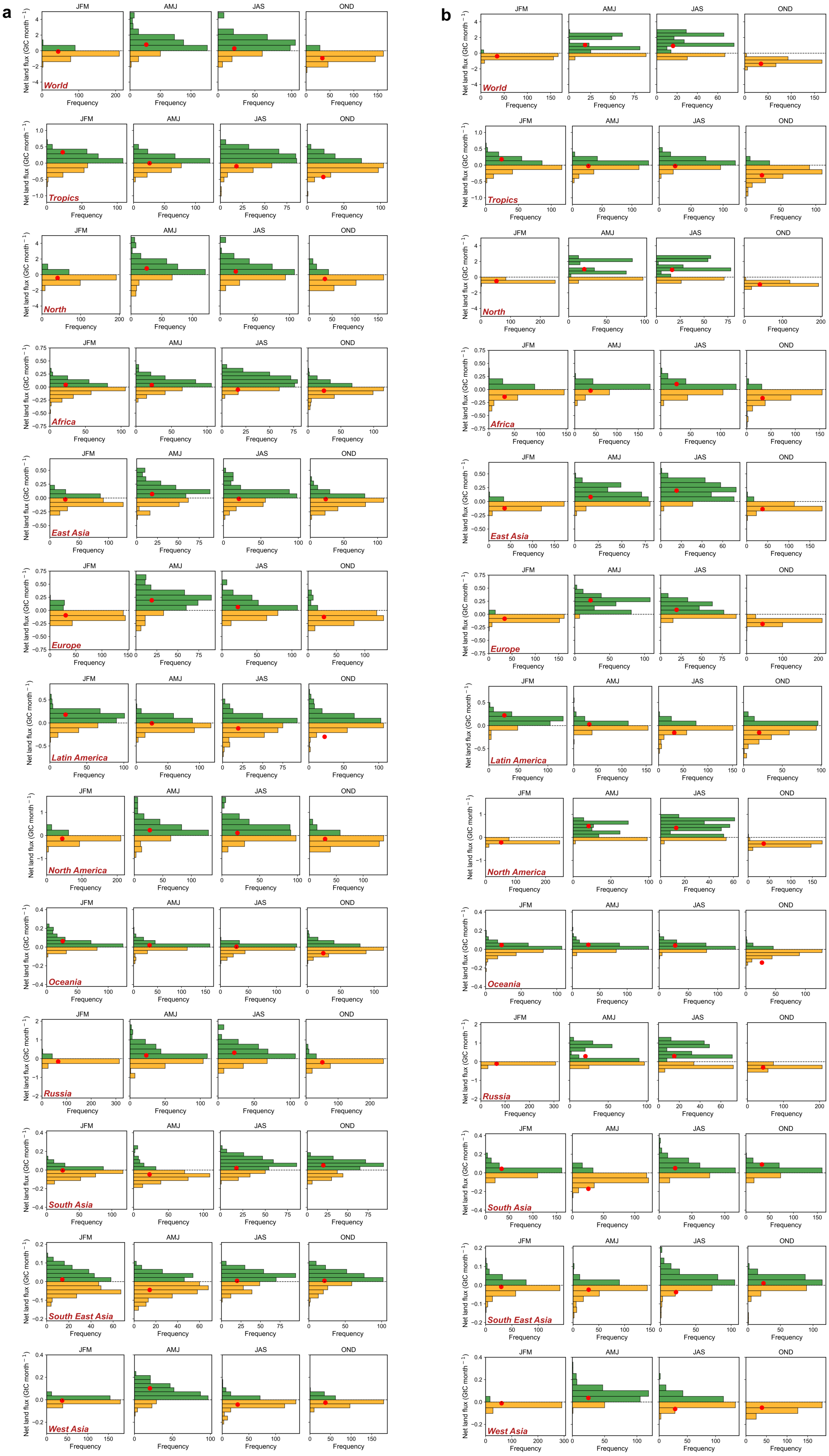
a
b
JFM
AMJ
JAS
OND
Net land flux (GtC month⁻¹)
Frequency
World
Tropics
North
Africa
East Asia
Europe
Latin America
North America
Oceania
Russia
South Asia
South East Asia
West Asia

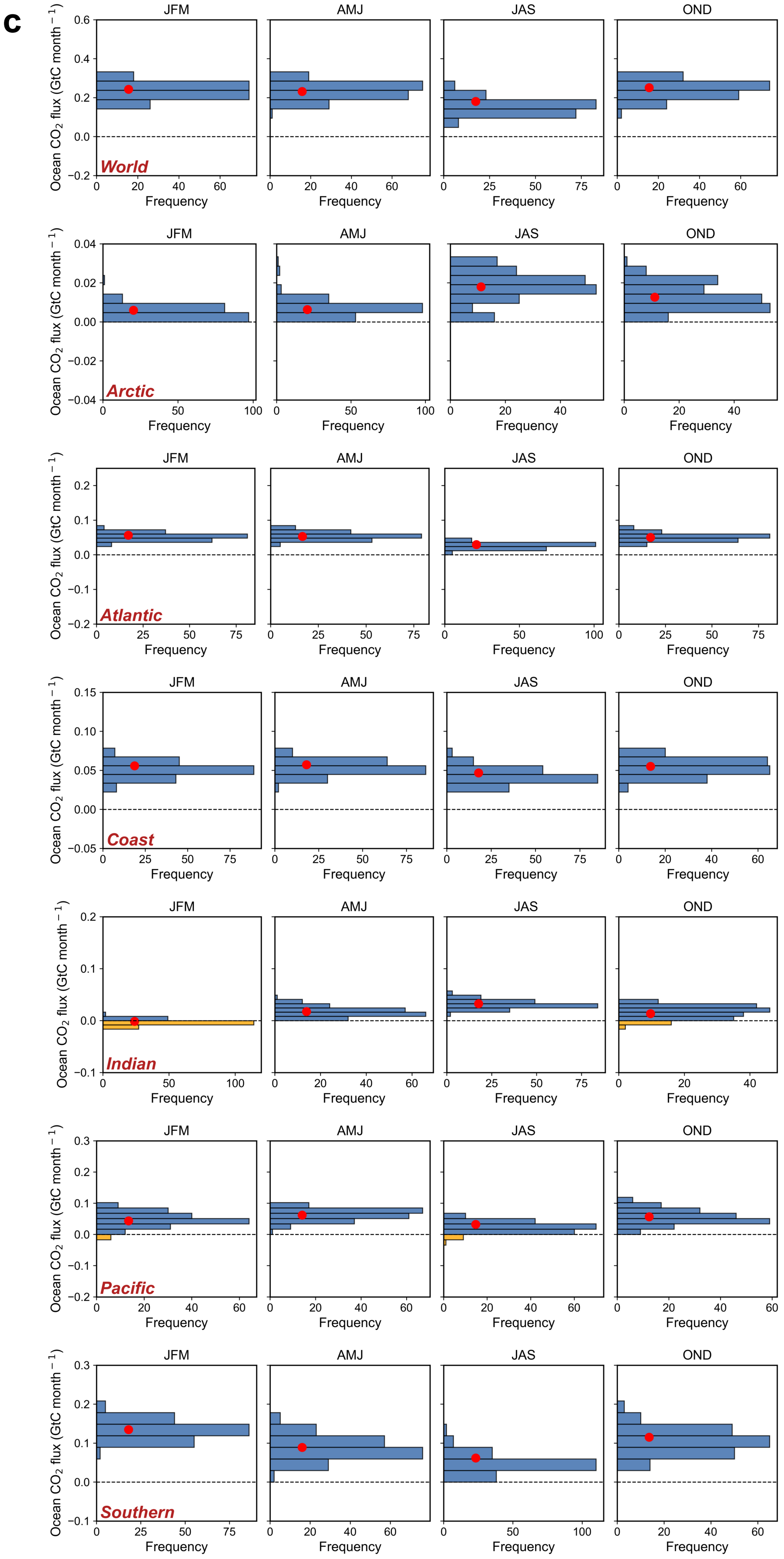
c
JFM
AMJ
JAS
OND
Ocean CO_2 flux (GtC month^{-1})
Frequency
World
Arctic
Atlantic
Coast
Indian
Pacific
Southern

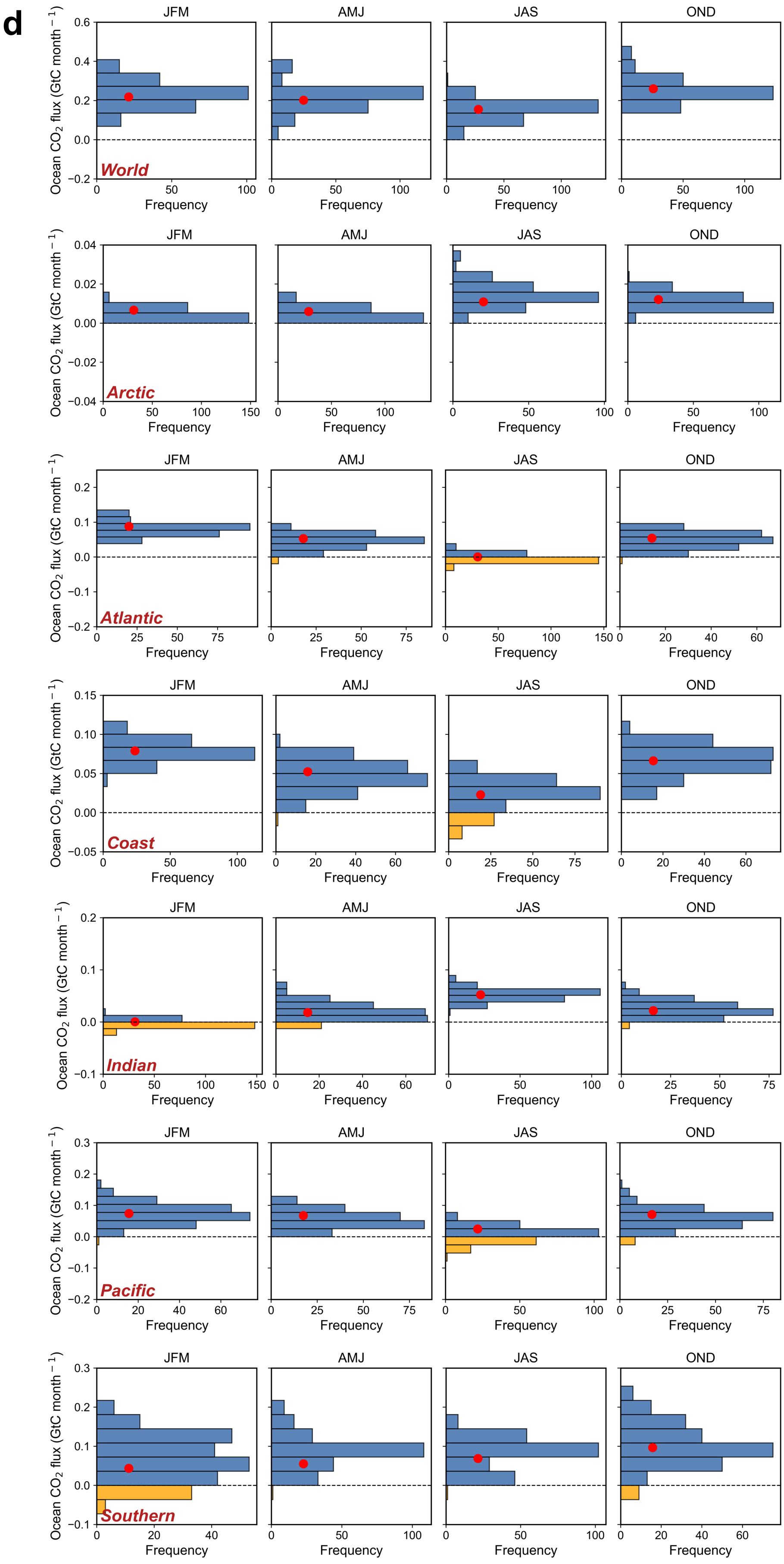
d
JFM
AMJ
JAS
OND
Ocean CO2 flux (GtC month−1)
Frequency
World
Arctic
Atlantic
Coast
Indian
Pacific
Southern

e

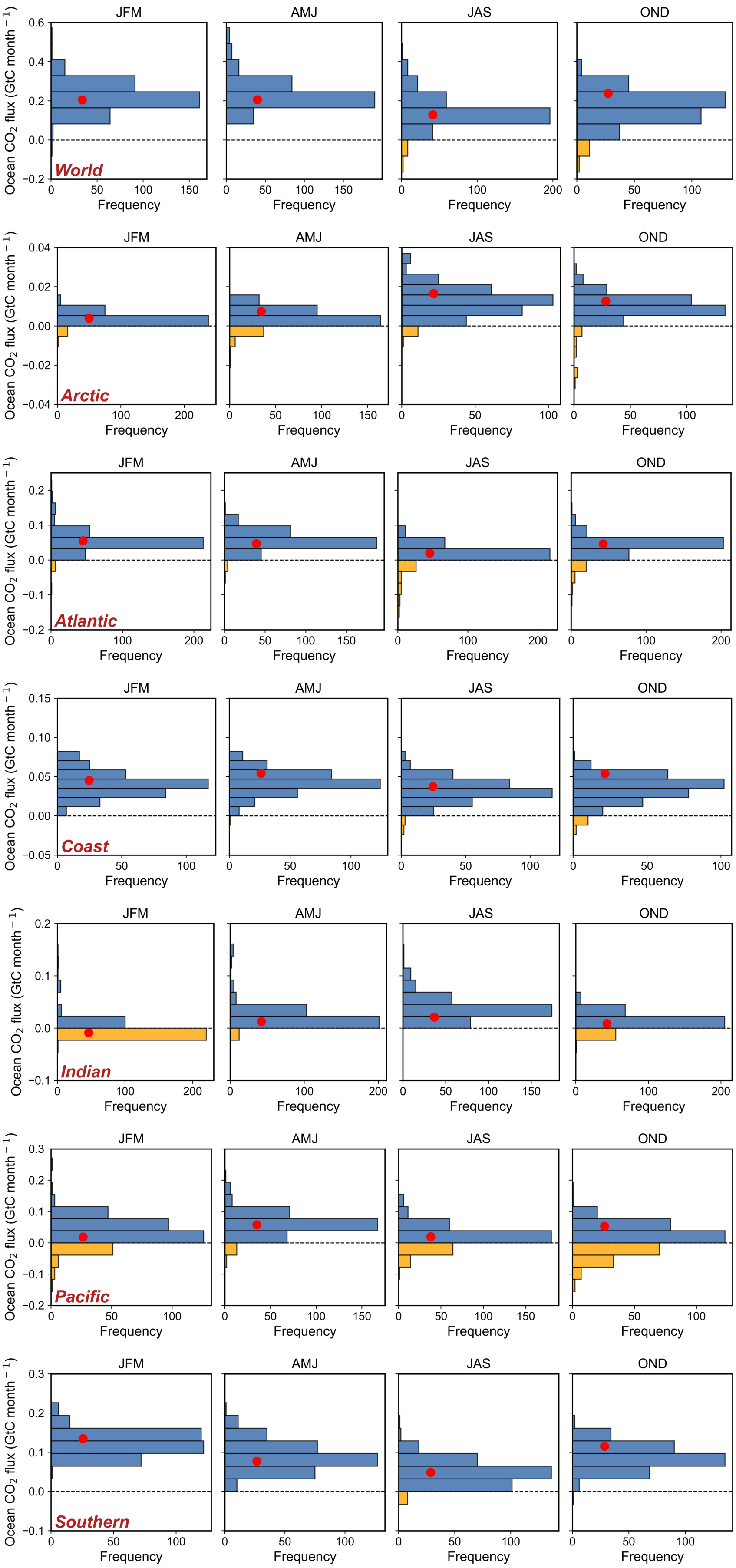
JFM
AMJ
JAS
OND
Ocean CO2 flux (GtC month −1)
Frequency
World
Arctic
Atlantic
Coast
Indian
Pacific
Southern

Supplementary Figure 4 Quarterly land and ocean fluxes for the RECCAP2 regions in 2023 and the distributions during 2015-2022. (a) Quarterly land flux in 2023 from 3 NRT DGVMs (red dots) for each RECCAP2 land region in the distribution of the flux from the DGVM models. (b) Quarterly land flux in 2023 from the OCO-2 inversion (red dots) for each RECCAP2 land region in the distribution of the flux from the inversion models. (c) Quarterly ocean flux in 2023 from the ocean data products emulators (red dots) for each RECCAP2 ocean region in the distribution of the flux from the data products models. (d) Quarterly ocean flux in 2023 from the ocean GOBMs emulators (red dots) for each RECCAP2 ocean region in the distribution of the flux from the GOBMs. (e) Quarterly ocean flux in 2023 from the OCO-2 inversion for each RECCAP2 ocean region in the distribution of the flux from the inversion models. Distributions are calculated during all previous years in the period 2015-2022. Positive values indicate flux from the atmosphere to the land or the ocean (carbon sink).

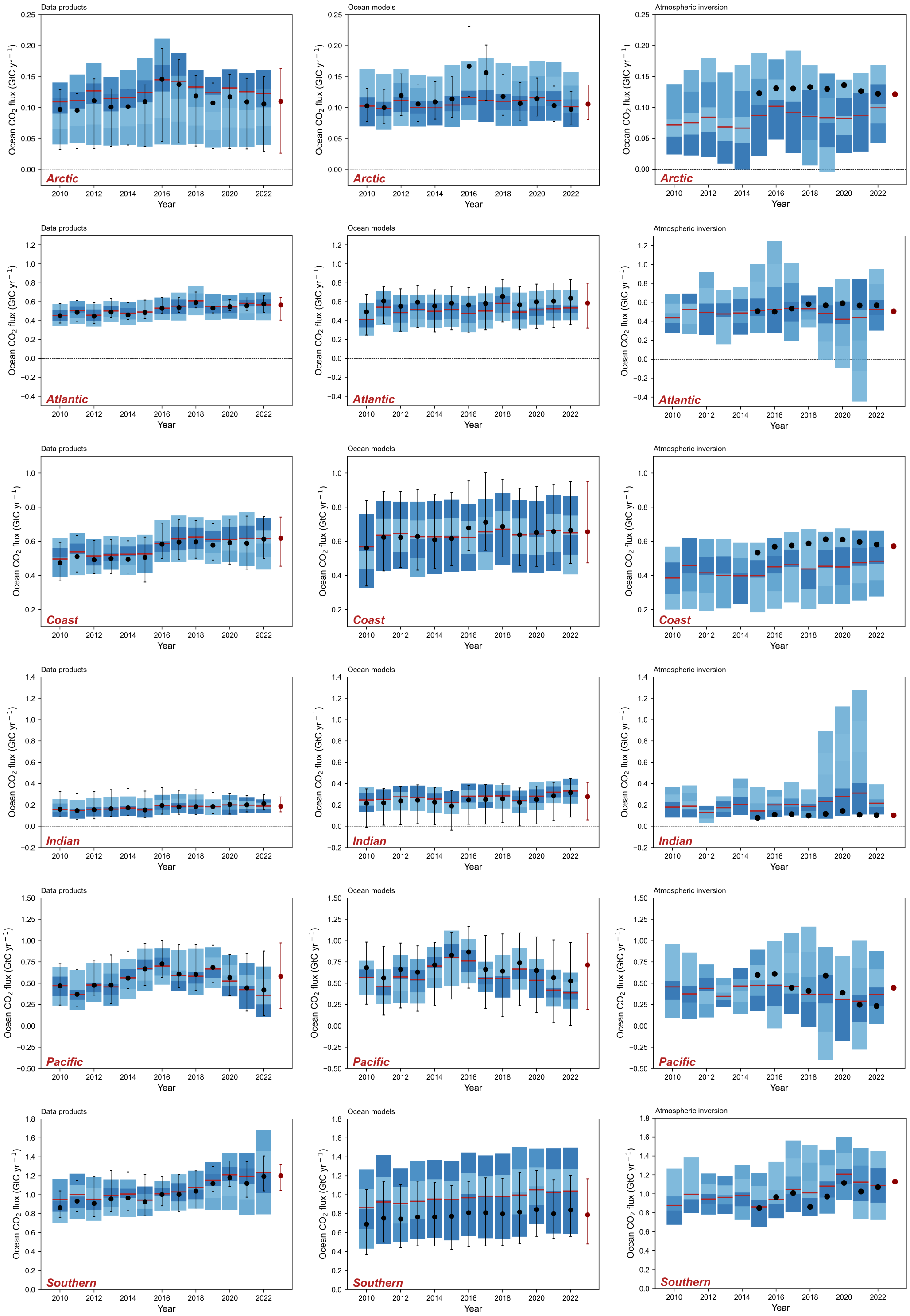

Supplementary Figure 5 Regional ocean fluxes for the RECCAP2 ocean regions from data products (left column), Global Ocean Biogeochemical Models (middle column) and inversion model (right column). The blue distribution is from the models used in latest Global Carbon Budget assessments (darker color means more models around a value). The median of models is the red line, the mean of the AI-based emulators or models used in this study is indicated by black dots and by a red dot in 2023. Positive values indicate flux from the atmosphere to the ocean (carbon sink).

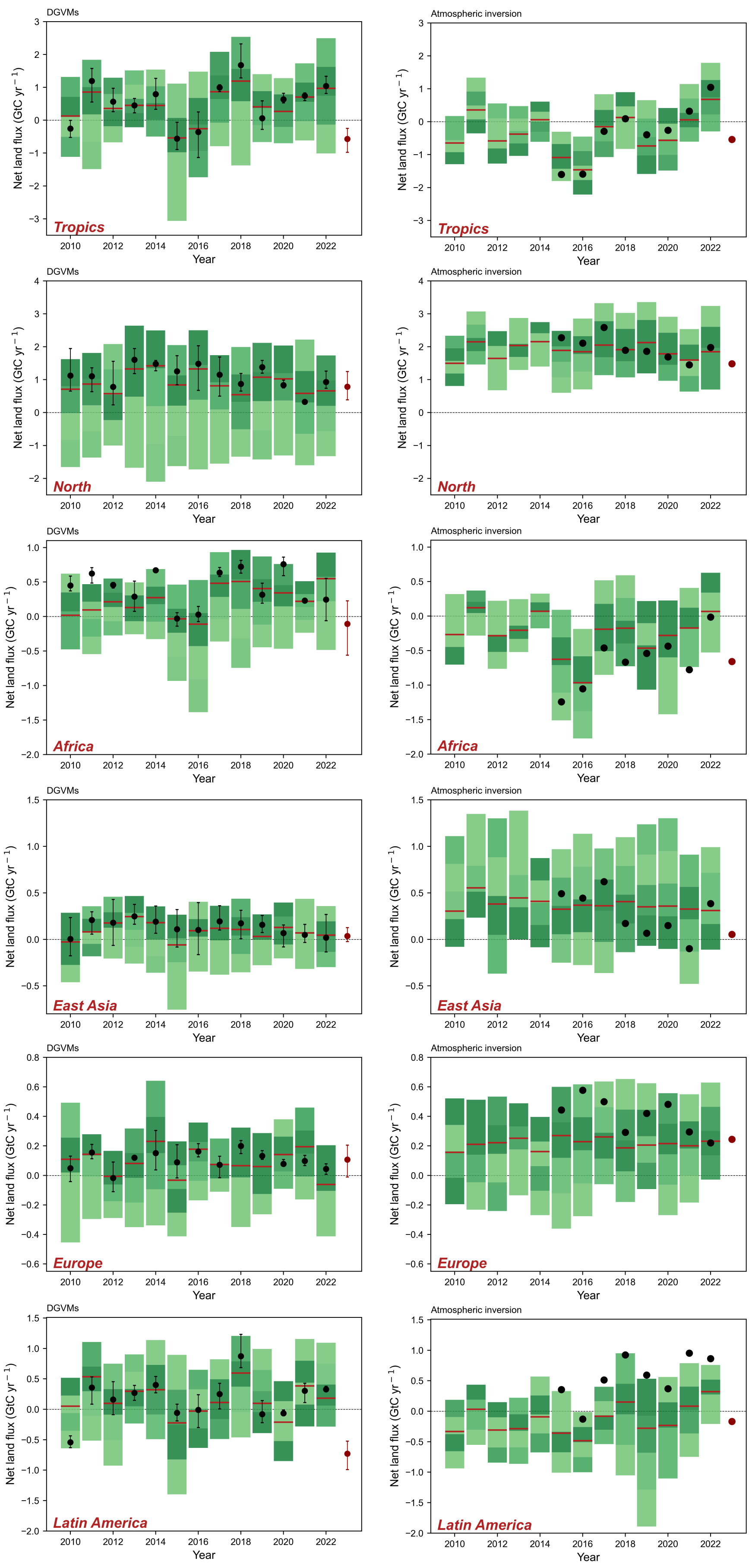

DGVMs
Atmospheric inversion
Net land flux (GtC yr^{-1})
Year
Tropics
North
Africa
East Asia
Europe
Latin America

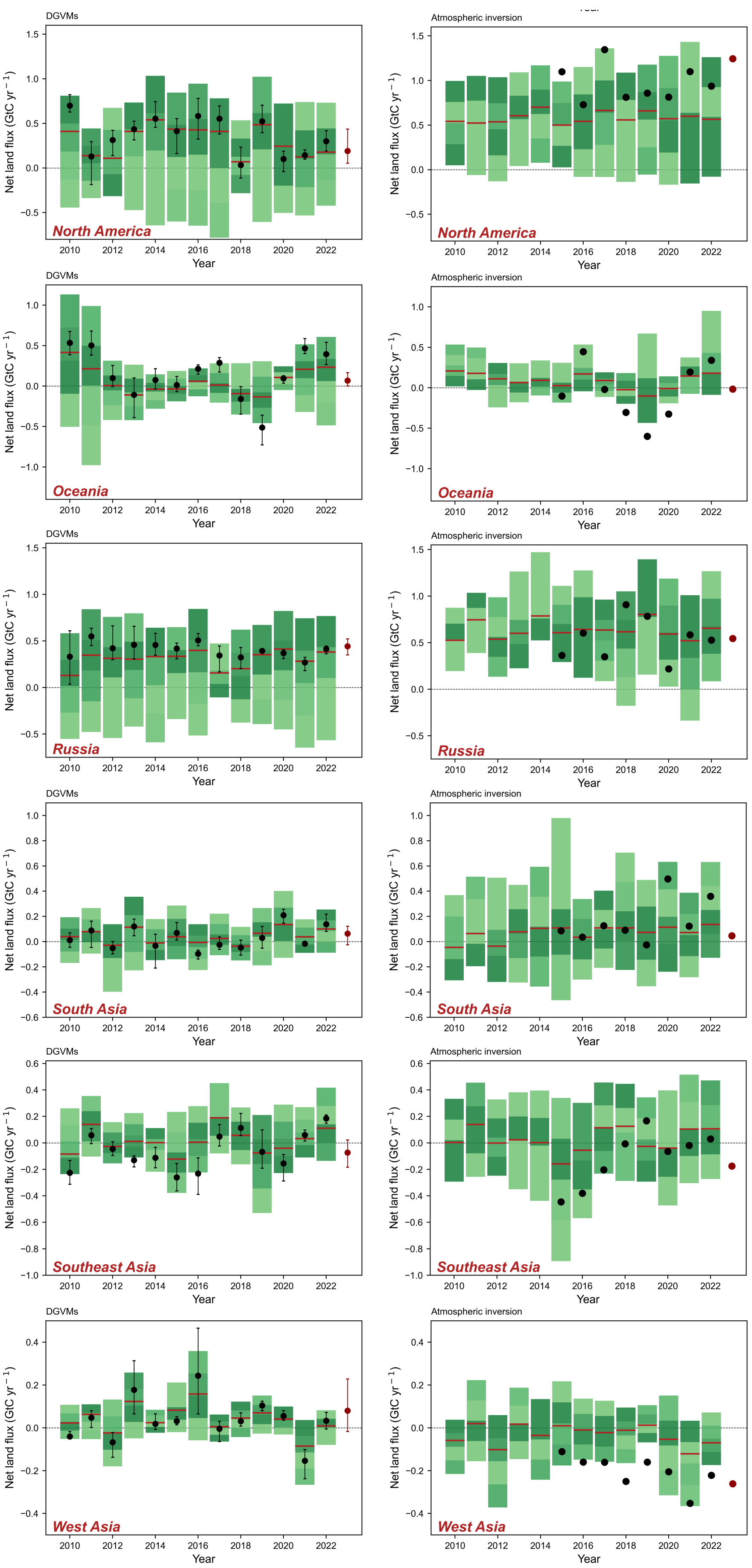

DGVMs
Atmospheric inversion
North America
Oceania
Russia
South Asia
Southeast Asia
West Asia
Net land flux (GtC yr^{-1})
Year
2010
2012
2014
2016
2018
2020
2022

Supplementary Figure 6 Regional land fluxes for the RECCAP2 land regions from DGVMs (left) and inversion models (right). The green color distribution is from the TRENDY and inversion models used in latest Global Carbon Budget assessments (darker color means more models around a value). The median of TRENDY and inversion models is the red line, the mean of the 3 DGVMs and OCO-2 inversion used in this study is indicated by black dots and by a red dot in 2023. Positive values indicate flux from the atmosphere to the land (carbon sink).

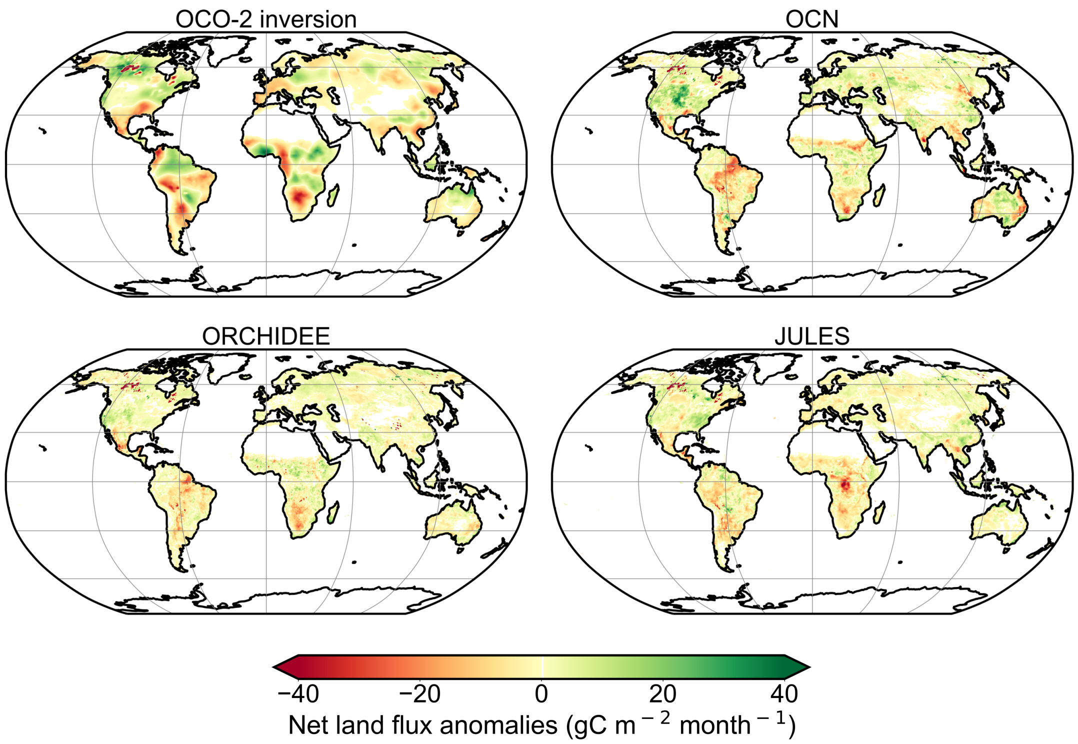

Supplementary Figure 7 Net land CO_2 flux anomalies in 2023 compared with the 2015-2022 average for the OCO-2 inversion and 3 near-real-time DGVMs. Positive values represent increased flux from the atmosphere to the land or ocean (carbon sink).

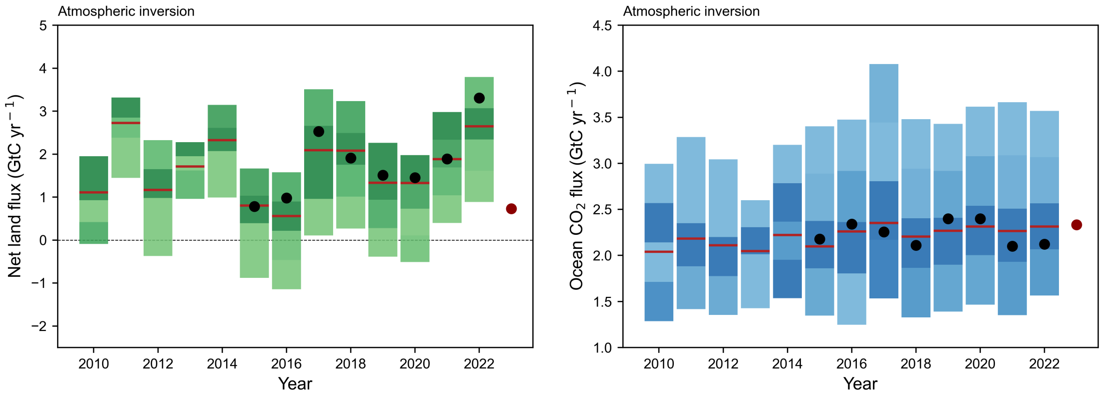

Supplementary Figure 8 Comparison of global land (left) and ocean (right) CO_2 fluxes between the OCO-2 inversion of this study (black dots, red dots for 2023) and the distribution of 14 inversion models (green bars for land, blue bars for ocean, with color intensity representing the number of models falling into each interval) used in ref.[7]

Supplementary Table 1 CO_2 fluxes (2010–2022 average and 2023) for each land RECCAP2 region from DGVMs and inversion methods (2010–2022 from GCB 2023; 2023 from NRT methods; Unit: GtC/yr).

Period	Methods	World	Tropics	North	Africa	East Asia	Europe	Latin America	North America	Oceania	Russia	South Asia	Southeast Asia	West Asia
2010 -2022 average	DGVMs	1.65 ± 2.76	0.45 ± 0.94	0.90 ± 2.42	0.24 ± 0.49	0.09 ± 0.36	0.09 ± 0.30	0.17 ± 0.69	0.31 ± 0.81	0.06 ± 0.23	0.30 ± 0.76	0.04 ± 0.18	0.01 ± 0.18	0.04 ± 0.07
	Inversions	1.67 ± 1.12	-0.33 ± 1.13	1.89 ± 0.39	-0.26 ± 0.71	0.38 ± 0.18	0.24 ± 0.41	-0.14 ± 0.46	0.58 ± 0.12	0.09 ± 0.19	0.64 ± 0.17	0.07 ± 0.12	0.03 ± 0.18	-0.04 ± 0.16
2023	DGVMs	0.14 ± 0.28	-0.57 ± 0.41	0.78 ± 0.46	-0.11 ± 0.45	0.04 ± 0.09	0.11 ± 0.12	-0.73 ± 0.26	0.19 ± 0.25	0.07 ± 0.10	0.44 ± 0.09	0.06 ± 0.09	-0.07 ± 0.11	0.08 ± 0.15
	Inversions	0.73 ± 0.30	-0.54 ± 0.20	1.48 ± 0.15	-0.66 ± 0.26	0.05 ± 0.17	0.24 ± 0.07	-0.17 ± 0.26	1.24 ± 0.19	-0.02 ± 0.10	0.54 ± 0.12	0.05 ± 0.22	-0.18 ± 0.22	-0.26 ± 0.17

Supplementary Table 2 CO_2 fluxes (2010–2022 average and 2023) for each ocean RECCAP2 region from bottom-up and inversion methods (2010–2022 from GCB 2023; 2023 from NRT methods; Unit: GtC/yr).

Period	Methods	World	Arctic	Atlantic	Coast	Indian	Pacific	Southern
2010-2022 average	GOBMs	2.48 ± 0.80	0.11 ± 0.06	0.51 ± 0.18	0.64 ± 0.22	0.27 ± 0.12	0.58 ± 0.24	0.97 ± 0.43
	Data products	2.46 ± 0.66	0.13 ± 0.09	0.52 ± 0.14	0.57 ± 0.13	0.18 ± 0.09	0.53 ± 0.27	1.06 ± 0.27
	Inversions	2.21 ± 0.17	0.08 ± 0.02	0.49 ± 0.07	0.44 ± 0.05	0.20 ± 0.11	0.40 ± 0.11	1.01 ± 0.20
2023	GOBMs	2.49 ± 0.79	0.11 ± 0.03	0.59 ± 0.26	0.66 ± 0.30	0.28 ± 0.22	0.71 ± 0.52	0.79 ± 0.38
	Data products	2.67 ± 0.60	0.11 ± 0.08	0.57 ± 0.16	0.62 ± 0.16	0.18 ± 0.09	0.58 ± 0.39	1.20 ± 0.16
	Inversions	2.33 ± 0.20	0.12 ± 0.20	0.51 ± 0.20	0.57 ± 0.20	0.10 ± 0.20	0.45 ± 0.20	1.13 ± 0.20

Supplementary Table 3 Quarterly differences of four versions of OCO-2 inversions in 2023 compared to quarterly means from 2015 to 2022 (Unit: GtC/yr).

Region	Quarter	Inversion without fires	Inversion with fires but regrowth	Inversion with fires without regrowth
World	Q1	0.066	0.070	0.030
	Q2	0.070	0.050	0.115
	Q3	-0.229	-0.243	-0.248
	Q4	-0.290	-0.243	-0.252
North America > 60°N	Q1	0.000	0.009	0.000
	Q2	0.018	0.022	0.019
	Q3	0.005	-0.009	0.007
	Q4	0.000	0.011	0.003
Europe > 60°N	Q1	0.001	0.000	0.002
	Q2	0.012	0.014	0.007
	Q3	-0.009	-0.007	-0.008
	Q4	-0.004	-0.004	-0.004
Siberia > 60°N	Q1	-0.001	-0.006	0.000
	Q2	-0.032	-0.034	-0.002
	Q3	0.038	0.051	0.041
	Q4	0.001	-0.003	-0.004

References

1. Lan X, Tans P, Thoning KW. *Trends in Globally-Averaged CO2 Determined from NOAA Global Monitoring Laboratory Measurements*.
2. Lan X, Keeling R. *Trends in Mauna Loa CO2 Determined from NOAA Global Monitoring Laboratory Measurements*.
3. Friedlingstein P, O'Sullivan M, Jones MW *et al.* Global Carbon Budget 2022. *Earth Syst Sci Data* 2022;**14**:4811–900.
4. Ke G, Meng Q, Finley T *et al.* Lightgbm: A highly efficient gradient boosting decision tree. *Adv Neural Inf Process Syst* 2017;**30**.
5. Kim Y. Convolutional Neural Networks for Sentence Classification. 2014.
6. Paszke A, Gross S, Massa F *et al.* Pytorch: An imperative style, high-performance deep learning library. *Adv Neural Inf Process Syst* 2019;**32**.
7. Friedlingstein P, O'Sullivan M, Jones MW *et al.* Global Carbon Budget 2023. *Earth Syst Sci Data* 2023;**15**:5301–69.
